# The majority of Canadians likely behaved as myopic rationalists rather than success-based learners when deciding on their first dose of COVID-19 vaccine

**DOI:** 10.3389/fpubh.2024.1406911

**Published:** 2024-07-24

**Authors:** Azadeh Aghaeeyan, Pouria Ramazi, Mark A. Lewis

**Affiliations:** ^1^Department of Mathematics and Statistics, Brock University, St. Catharines, ON, Canada; ^2^Department of Mathematics and Statistics and Department of Biology, University of Victoria, Victoria, BC, Canada

**Keywords:** vaccination, decision-making strategies, vaccine promotion, mechanistic model, Canada

## Abstract

**Introduction:**

Successful vaccine promotion communication strategies require knowing how eligible recipients will respond to the opportunity to get vaccinated. Two main classes of recipients are myopic rationalists, those who receive a dose of vaccine only if it maximizes their own instant benefit and if so, do it as soon as possible, and success-based learners, those who learn from others that they perceive to be most successful.

**Methods:**

A recent study models these two decision-making types, and estimates the population proportion of myopic rationalists in each U.S. state. In this report, we fit a similar model to data on COVID-19 vaccine uptake across the Canadian provinces and territories.

**Results:**

We estimated that 64% of Canadians behaved as myopic rationalists in taking the first dose of a COVID-19 vaccine, compared to an estimated 47% in the United States. Among the provinces, the lowest proportion of myopic rationalists was 0.51 in Saskatchewan, while the highest was 0.74 in Prince Edward Island. The correlation analysis suggested a positive correlation between the proportion of myopic rationalists and the average age across the Canadian provinces (Pearson-*r* = 0.71).

**Discussion:**

Canadian health management may benefit from these results in tailoring the vaccine promotion communication strategies.

## Introduction

COVID-19 vaccination programs and the subsequent responses of individuals to the opportunity to be vaccinated have accentuated the need for more effective communication strategies. Several studies have been devoted to reporting the relation between the final decision of individuals toward vaccination and a variety of factors, ranging from political partisanship (1) and employment rate to ethnicity (2), locale of residence (3), and so on. Other studies have tried to find the significant factors impacting individuals' attitude toward vaccination (4, 5). However, one crucial yet often neglected factor in these studies is the vaccination rate, which, aside from logistical considerations, seems to be closely tied to human decision-making strategies.

Indeed, it has been reported in a variety of contexts that individuals vary in their decision making strategies (6–8). Two ends of the spectrum of the decision-making types are *myopic rationalists*, those who go for a decision that maximizes their perceived instant benefit, and *success-based learners*, those who decide based on learning from others and their satisfaction with the decision they made. In evolutionary game theory, the former is also known as best-responders (9, 10), and the latter is known as imitators (11, 12). In the context of vaccination, the individuals' decision-making strategies impact the time they need to make their minds and get the vaccine. Furthermore, the information individuals seek may depend on their decision-making strategies (7). Hence, health authorities seeking to promote vaccination may benefit from knowing the proportions of these two decision-making types. This issue, however, has not received much attention, especially in the context of vaccination.

Recently, we proposed a mechanistic model that allows for the differences in human decision-making strategies (13). The model was constructed on the assumption that non-vaccine refusers are either myopic rationalists or success-based learners. From fitting the model to the datasets on COVID-19 vaccination across the U.S. states, we showed almost equal numbers of American myopic rationalists and success-based learners in deciding over taking the first dose of a COVID-19 vaccine. There was a huge variation in the proportion across the states of the U.S. These results prompted us to ask whether a similar pattern in human decision-making strategies exists in other nations such as Canada.

In this study, we estimate the proportion of residents in each jurisdiction of Canada who behaved as myopic rationalists in taking the first dose of a COVID-19 vaccine. We then investigate the linear correlation between the estimated proportion of myopic rationalists and some possible explanatory variables such as average age, employment rate, vaccination coverage, and the proportion of residents with graduate studies.

## Materials and methods

### Data

We used the temporal data on the number of COVID-19 vaccine doses delivered to Canadian provinces and territories (14) to calculate the cumulative number of available doses for the first shot. The data on new confirmed cases, deaths, and administered vaccine doses were obtained from (15). The procedure described in (13) was used to clean the data. Please refer to Supplementary material for additional details on data cleaning.

We additionally included possible explanatory variables for success-based learning, such as the proportion of employed residents (16), average income of residents (17), average age of residents (18), proportion of visible minorities (19), and proportion of residents with graduate degree (20). To include the possible variation in the perception of the residents of each jurisdiction in Canada toward COVID-19 vaccine associated side effects, we used the results of a longitudinal study (21). Details can be found in Supplementary material. We estimated the proportion of vaccine refusers by the proportion of people who remained unvaccinated as of June 2023–the most recent date for which the vaccination data was available.

### Model formulation

Vaccination dynamics are complex and are affected by changing population sizes as well as heterogeneous mixing. However, for the purpose of modeling the behavioral aspects of vaccine decision-making we made some simplifying assumptions as given in (13). Within each jurisdiction and over the time of vaccination we assume that populations are of fixed size and well mixed. In most cases the vaccination of individuals under the age of 12 would require guardian consent (22). Hence, we exclude them to avoid double counting the decisions made by their guardians.

Not all eligible individuals will receive a vaccine. Indeed, some individuals, whom we refer to as *vaccine refusers*, will not receive a dose of a COVID-19 vaccine under any circumstances. The remaining population, *N*_*n*_, will have a continuum of different possible behavioral responses to vaccination. However, we approximate these responses as falling into one of two behavioral groups; myopic rationalists with population size α_1_*N*_*n*_ and success-based learners with population size (1 − α_1_)*N*_*n*_.

Myopic rationalists and success-based learners are assumed to decide on vaccination based on the perceived payoff gain for vaccination, Δπ(*t*). In (23), it was assumed that the perceived payoff gain for whole-cell pertussis vaccination is shaped by the perceived probability of significant morbidity from vaccine and the perceived risk of infection when a person is not vaccinated. We additionally consider the risk of death due to COVID-19 and the perceived socio-economic benefits of vaccination in a disease-free situation. The socio-economic benefits of vaccination include policies that differentiate between vaccinated and unvaccinated individuals, such as allowing entry to public events, gatherings, and workplaces.

We model the impact of epidemiological conditions on the payoff gain as the summation of the perceived risk reductions in morbidity, *c*_*c*_*C*(*t*)/*N*, and mortality, *c*_*d*_*D*(*t*)/*N*, due to COVID-19 obtained from a dose of COVID-19 vaccine. Here *C*(*t*) and *D*(*t*), respectively, represent the weekly number of confirmed COVID-19 cases and deaths due to COVID-19, *N* denotes the total population size, and *c*_*c*_ and *c*_*d*_ represent the perceived cost reduction in morbidity and mortality, respectively. The perceived risk of suffering from vaccine-associated side effects is formulated as *c*_*v*0_*f*(*t*), where *c*_*v*0_ represents the perceived cost of vaccine associated side effects and *f*(*t*) represents the commonality of concerns about vaccine side effects at week *t*. We estimate *f*(*t*) based on the proportion of responders concerned about COVID-19 vaccine side effects in a longitudinal study conducted by Impact Canada (21). The perceived socio-economic benefits of receiving a dose of COVID-19 vaccine is modeled by a free parameter $c_{\bar{v}}$. We further assume that the payoff gain for vaccination is the sum of these factors, resulting in the following formula:


(1) $$\begin{array}{l} \Delta \pi \left(t\right)=c_{\bar{v}}-c_{v0}f\left(t\right)+c_{c}\frac{C\left(t\right)}{\text{N}}+c_{d}\frac{D\left(t\right)}{\text{N}}. \end{array}$$

In our model, no one would go for vaccination if the perceived vaccination payoff defined in Equation 1 is negative. If it is positive, then the myopic rationalists would get vaccinated as soon as possible. The success-based learners, however, would be influenced by both the size of the perceived vaccination gain and the vaccination coverage. Under no vaccine doses limitation, all vaccine seekers would become vaccinated by a maximum rate of vaccination κ. When this is not the case, we assume that the available vaccine doses are randomly distributed among the vaccine seekers. Hence, the evolution of vaccine uptake when the perceived payoff gain for vaccination is positive can be summarized as follows:


$$\begin{array}{l} \overset{\begin{array}{c} \text{rate of change of}\\ \text{vaccinated learners} \end{array}}{\overset{︷}{\dot{L}\left(t\right)}}=\overset{\begin{array}{c} \text{maximum}\\ \text{rate of}\\ \text{vaccination} \end{array}}{\overset{︷}{\kappa }}\overset{\begin{array}{c} \#\text{of unvaccinated}\\ \text{learners} \end{array}}{\overset{︷}{\left(\left(1-\alpha_{1}\right)N_{n}-L\left(t\right)\right)}}\overset{\begin{array}{c} \text{proportion of}\\ \text{vaccinated people} \end{array}}{\overset{︷}{\frac{L\left(t\right)+M\left(t\right)}{N}}}\overset{\begin{array}{c} \text{dimensionless}\\ \text{payoff gain} \end{array}}{\overset{︷}{\sigma \Delta \pi \left(t\right)}}\times \\ \;\min\left\{1,\frac{\overset{\#\text{of available doses}}{\overset{︷}{v\left(t\right)-L\left(t\right)-M\left(t\right)}}}{\underset{\text{vaccine demand}}{\underset{︸}{\left(\left(1-\alpha_{1}\right)N_{n}-L\left(t\right)\right)\frac{L\left(t\right)+M\left(t\right)}{N}\sigma \Delta \pi \left(t\right)+\left(\alpha_{1}N_{n}-M\left(t\right)\right)}}}\right\},\\ \overset{\begin{array}{c} \text{rate of change of}\\ \text{vaccinated rationalists} \end{array}}{\overset{︷}{\dot{M}\left(t\right)}}=\kappa \overset{\begin{array}{c} \#\text{number of unvaccinated}\\ \text{rationalists} \end{array}}{\overset{︷}{\left(\alpha_{1}N_{n}-M\left(t\right)\right)}}\times \\ \;\min\left\{1,\frac{v\left(t\right)-L\left(t\right)-M\left(t\right)}{\left(\left(1-\alpha_{1}\right)N_{n}-L\left(t\right)\right)\frac{L\left(t\right)+M\left(t\right)}{N}\sigma \Delta \pi \left(t\right)+\left(\alpha_{1}N_{n}-M\left(t\right)\right)}\right\}, \end{array}$$

where *v*(*t*) is the accumulated number of delivered doses up to week *t* and σ is the constant of proportionality. If the perceived payoff gain for vaccination, Δπ, is negative, then no one will be vaccinated yielding $\dot{L}\left(t\right)=\dot{M}\left(t\right)=0$.

### Parameter estimation and correlation analysis

The list of free parameters, variables, and fixed parameters is given in Table 1. Following (13), we assumed perceived effectiveness of 100% for a dose of a COVID-19 vaccine in preventing death, with the perceived monetary equivalent value of life set at one million dollars, representing the highest possible value (26). This results in *c*_*d*_ = 1. In (13), we observed that considering σ as a free parameter instead of fixing it at 1 did not alter the estimated value of the parameter of interest α_1_ considerably, but it introduced variability. Therefore, we set σ equal to 1. Parameters κ, α, *c*_*v*0_, $c_{\bar{v}}$, and *c*_*c*_, were capped at 10, 1, 1, 0.1, and $\underset{t}{\min}\frac{D\left(t\right)}{C\left(t\right)}$, for a nonzero *D*(*t*), respectively.

**Table 1:** List of parameters and variables.

**Parameter/variable**	**Symbol**	**Unit**	**Source**
Population of individuals aged 12 and above	*N*	Number	(24)
Total population	*N*	Number	(24)
Population of non-vaccine refusers	*N*_*n*_	Number	(25)
Number of new confirmed cases in week *t*	*C*(*t*)	Number	(15)
Number of new confirmed deaths in week *t*	*D*(*t*)	Number	(15)
Accumulated number of delivered doses up to week *t*	*v*(*t*)	Number	(14)
Estimated proportion of individuals concerned about vaccine associated side effects at week *t*	*f*(*t*)	Dimensionless	(21)
Number of vaccinated myopic rationalists up to week *t*	*M*(*t*)	Number	–
Number of vaccinated success-based learners up to week *t*	*L*(*t*)	Number	- -
Perceived payoff gain for vaccination	Δπ	$ M	–
Proportion of myopic rationalists among non-vaccine refusers	α_1_	Dimensionless	To be estimated
Population proportion of myopic rationalists	α	Dimensionless	*N*_*n*_α_1_/*N*
Maximum rate of vaccination	κ	Week^−1^	To be estimated
Perceived socio-economic benefits of vaccination in the absence of confirmed cases or deaths	$c_{\bar{v}}$	$ M	To be estimated
Perceived cost of vaccine associated side effects	*c*_*v*0_	$ M	To be estimated
Perceived cost reduction in morbidity due to COVID-19 obtained from a dose of vaccine	*c*_*c*_	$ M	To be estimated
Perceived cost reduction in mortality due to COVID-19 obtained from a dose of vaccine	*c*_*d*_	$ M	Set to 1
Constant of proportionality	σ	$\frac{1}{\$\text{M}}$	Set to 1

The notation *$* M stands for million dollars.

The model was fit to the data on the number of new vaccinated individuals per week, i.e., *n*_*v*_[*k*] = *N*_*v*_[*k*]−*N*_*v*_[*k*−1] with *n*_*v*_[0] = *N*_*v*_[0]. The error function was the residual sum of squares, $\Sigma_{k}||n_{v}\left[k\right]-{\hat{n}}_{v}\left[k\right]|{|}^{2}$ where the estimated number of individuals receiving their first dose of COVID-19 vaccine at time *k* was denoted by ${\hat{n}}_{v}\left[k\right]$. We used Python and the dual annealing optimization algorithm (27) to minimize the error function. Following (13), the control parameters were set as follows *initial_temp* = 50, 000 and *maxiter* = 2, 000. We ran the algorithm with five distinct seeds: 2024, 2025, 2026, 2027, and 2028, and recorded the estimated parameters. For each Canadian jurisdiction, we selected the set of parameters corresponding to the least error function.

The 95% confidence intervals were calculated using the residual non-parametric bootstrapping approach detailed in (13). The simulation results showed negligible changes in terms of the residual sum of squares when we substituted a free parameter within the range of [−1, 1] for $c_{\bar{v}}-c_{v0}f\left(t\right)$ (Supplementary Tables S1, S2). As a result, we opted for the simpler case. This modification, however, did not impact the point estimate of α_1_ as the relative change in the estimated α_1_ was < 5% in all provinces.

The linear correlations between possible explanatory variables and the estimated proportion of people who behaved like myopic rationalists in taking the first dose of COVID-19 vaccine, $\alpha =\frac{\alpha_{1}N_{n}}{N}$, were investigated. There were sufficient data for the 10 Canadian provinces, but not for the three territories on the possible explanatory variables. We, hence, only considered Canadian provinces in correlation analysis.

## Results

**Figure 1:**
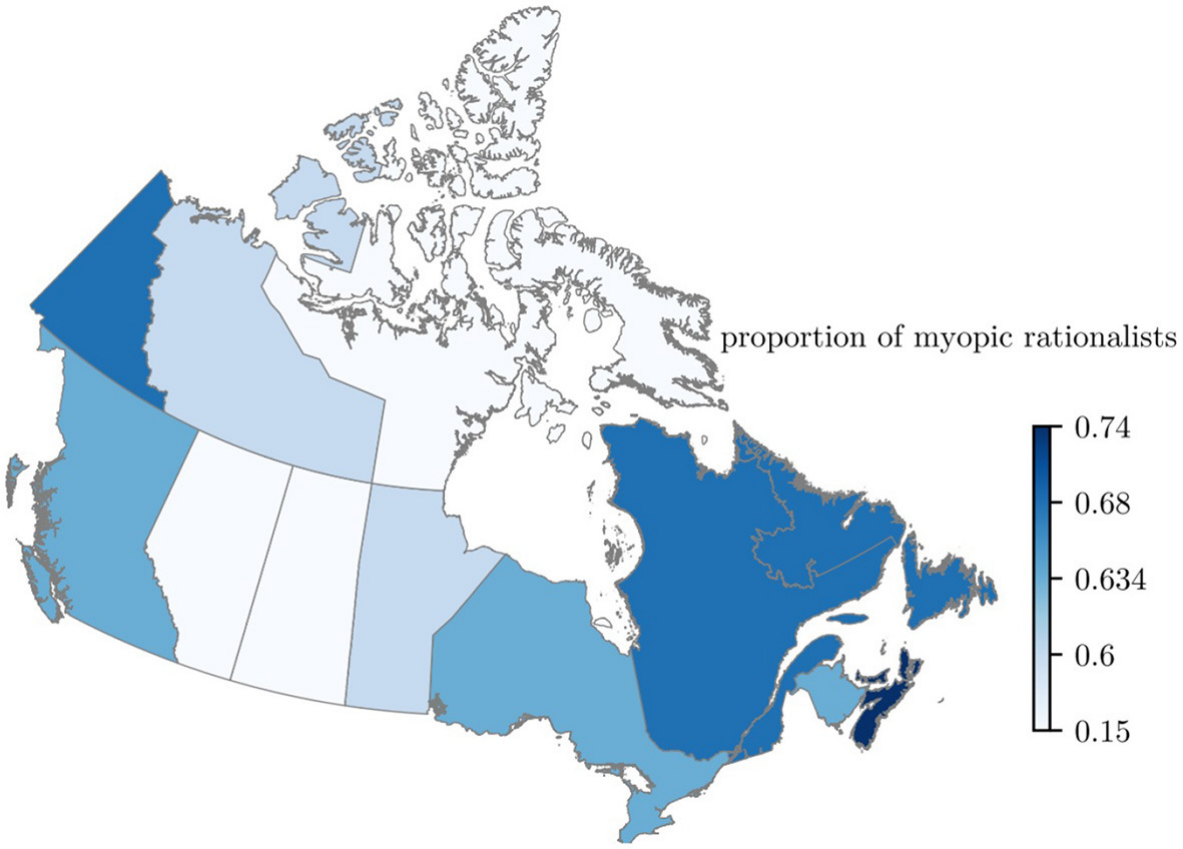
Map of Canada colored based on the estimated proportion of individuals behaving as myopic rationalists in receiving the first dose of a COVID-19 vaccination. Lighter colors show a lower proportion of myopic rationalists. The proportion of myopic rationalists aged 12 and above was estimated by fitting the proposed model in (13) to data on the weekly count of individuals receiving the first dose of a COVID-19 vaccine. The nation-wide estimated proportion of myopic rationalists was 0.64. There was a high degree of variation across the 13 jurisdictions, i.e., 0.18 for Nunavut to 0.74 for Prince Edwards Island.

By fitting the model to Canadian datasets on COVID-19 vaccine uptake, it was estimated that 64% of Canadian residents aged 12 and above behaved as myopic rationalists in taking the first dose of a COVID-19 vaccine (α = 0.64), 26% behaved as success-based learners, and the remaining 10% were vaccine refusers, i.e., they have remained unvaccinated as of June 18, 2023–the most recent date for which vaccination data was available (Figure 1).

**Figure 2:**
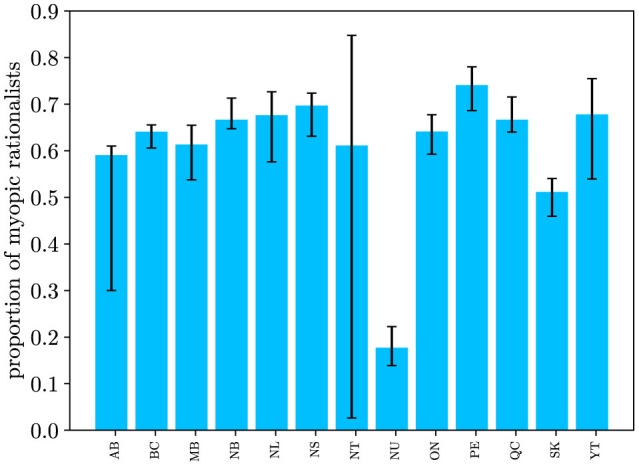
Estimated 95% confidence interval of the proportion of myopic rationalists for the Canadian provinces and territories. The estimated confidence interval of α for the Northwest Territories indicates a high degree of uncertainty. There are significant differences between the population proportions of myopic rationalists in Alberta and Saskatchewan compared to other Canadian provinces.

Except for the Northwest Territories and Alberta, the width of the 95% confidence interval for the estimated proportion of myopic rationalists was 0.22 or less. The estimated confidence interval for Alberta was [0.30, 0.61] and that of the Northwest Territories was [0.03, 0.85] (Figure 2).

**Figure 3:**
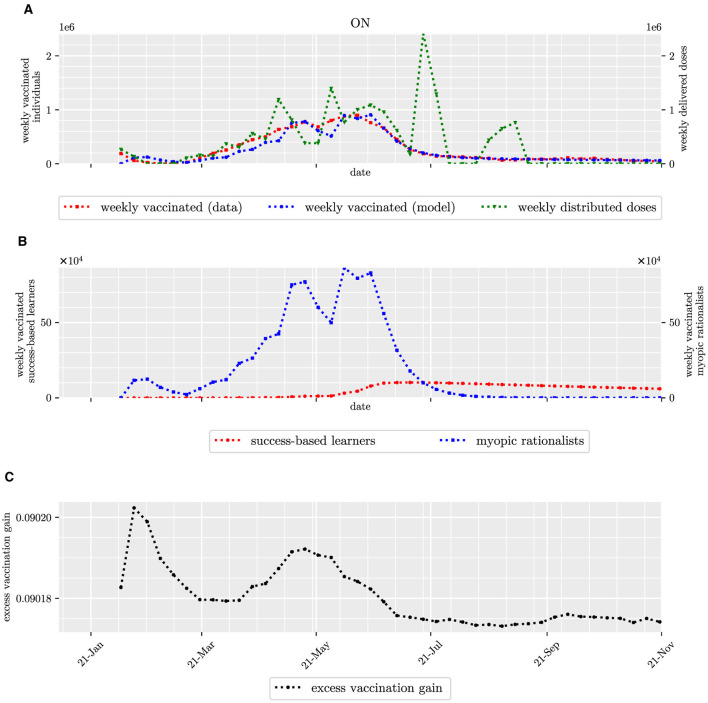
Count of individuals vaccinated weekly in Ontario, including myopic rationalists and success-based learners, and the perceived excess payoff of receiving a dose of COVID-19 vaccine. Top panel **(A)** depicts the count of individuals vaccinated weekly in red, its estimation in blue, and the count of weekly delivered doses in green. Second row **(B)** depicts the estimated count of myopic rationalists and success-based learners vaccinated weekly in blue and red, respectively. The last row **(C)** depicts the perceived excess payoff of receiving a dose of a COVID-19 vaccine over time.

During the first months of the vaccine roll-out, the trend of vaccine uptake followed the distribution pattern of vaccine doses (Figure 3A). As of August 2021, the vaccination coverage among eligible myopic rationalists who were residents of Ontario reached 100% (Figure 3B).

Our results indicated significant differences between the estimated proportions of myopic rationalist among the residents of Saskatchewan and Alberta and those of other Canadian provinces as well as Canada as a whole. More specifically, the upper limits of 95% confidence intervals of α in Saskatchewan and Alberta were lower than the estimated proportions of other provinces as well as across Canada.

Based on our fitting results, as of November 2021, every myopic rationalist in Canada had received at least one dose of a COVID-19 vaccine. The vaccination coverage among the success-based learners, however, varied across the jurisdictions ranging from 36.6% in Alberta to 96.1% in Nunavut (Supplementary Table S3). Nationwide vaccination coverage was 63% among Canadian success-based learners.

The estimated population proportion of myopic rationalists was positively correlated with the average age in the Canadian provinces (Pearson-*r* = 0.70) and the proportion of senior residents (Pearson-*r* = 0.71). However, the proportion was negatively correlated with the employment rate in the Canadian provinces (Pearson-*r* = −0.56). The proportion was not highly correlated with the vaccination coverage as of June 2023 (Pearson-*r* = 0.34), the proportion of residents with graduate studies (Pearson-r = 0.22), income per capita (Pearson-*r* = −0.11), and poverty rate (Pearson-*r* = 0.09).

## Discussion

In this study, we modeled that those Canadians who eventually received at least one dose of a COVID-19 vaccine behaved as either a myopic rationalist or a success-based learner. We then estimated the proportions of these two types by applying the model and the methodology proposed in (13). Canada-wide population proportions of myopic rationalists and success-based learners were estimated 0.64 and 0.26, respectively.

It is acknowledged that these two decision-making types are the two ends of the spectrum of reliance on others' behavior. Myopic rationalists are those who only take into account the factors influencing their perceived payoff gains, and their decisions are not informed by those of others. On the other hand, success-based learners learn via interactions with others; they need to meet others, compare their own perceived payoffs with those of others, and then they follow the decisions of others with a probability proportional to the differences in the perceived payoffs. There could, however, be some intermediate decision-making types whose decisions about vaccination are simultaneously influenced by their social interactions and their own perceived instant benefit of vaccination (28).

Our simulation results suggested that the vaccination uptake over time can be sufficiently explained by two graphs: one representing the vaccination progress of those who preferred to wait to hear from others and not rush into vaccination, and the other accounting for that of individuals who would get vaccinated as soon as they found it beneficial. In this study, the former behavior was that of success-based learners and the latter was that of myopic rationalists. Investigating a more complete mechanistic model that allows for an additional intermediate group of decision-makers is left for future work.

Based on our proposed model for the perceived payoff gain for vaccination and the fitting results, all myopic rationalists and success-based learners would receive a dose of a COVID-19 vaccine. However, these two types of decision-makers would vary in the required time to decide. Our results indicated that the vaccination coverage among myopic rationalists was 100%. This result is consistent with their decision-making strategy: they consider vaccination only if the perceived payoff for vaccination is greater than that of remaining unvaccinated. If it is, they get vaccinated immediately, with vaccine availability being the only limiting factor. There was, however, a high degree of variation in the vaccination coverage among success-based learners across the Canadian provinces as of November 2021–the last date of fitting. This suggests that the majority of the vaccine promotion efforts should go to success based learners.

Success-based learners of Alberta and Saskatchewan had the lowest vaccination coverage (Supplementary Table S3). The health management of these two provinces might benefit from this result to tailor the future vaccine promotion programs to success-based learners. In particular, success-based learners are social learners and tend to imitate the decisions of the perceived most successful individuals in the populations. For example recruiting social media influencers to share their positive experiences of receiving a dose of vaccine may increase the vaccination coverage among success-based learners.

Incorporating a time-varying perception of vaccine associated side effects did not improve the fitting results (see Supplementary Tables S1, S2). This could be attributed to the absence of a consistent pattern in the proportion of people concerned about vaccine side effects. Another potential reason could be the sparsity of data, with only five data points recorded over the time period from December 2020 to June 2021 (Supplementary material).

At least half of the population of each province was estimated to be myopic rationalists, with the lowest proportion in Saskatchewan (0.51). This contrasts with the U.S., where the state of Mississippi had the lowest proportion of myopic rationalists, at 0.31. The variation in the estimated proportion of myopic rationalists across Canadian provinces ranged from 0.51 for Saskatchewan to 0.74 for Prince Edward Island. This variation was lower than the variation observed in the U.S. states (0.31 for the state of Mississippi to 0.76 for the state of Vermont) (13).

Currently, Saskatchewan and Alberta are the only provinces in Canada with a majority of House of Commons seats registered as Conservative (29). The significant lower differences between the estimated proportions of myopic rationalists in these two provinces and the other provinces of Canada are consistent with the reported results in the U.S., where a high correlation between the proportion of success-based learners and Republicans was reported (13).

According to our proposed model for the perceived payoff gain for vaccination and the fitting results, all myopic rationalists and success-based learners would eventually get vaccinated. However, myopic rationalists would get vaccinated as soon as they found it beneficial, i.e., as soon as the perceived payoff gain for vaccination became positive. Based on the fitting results, the payoff gain for vaccination was positive from the start of vaccine roll-out in each Canadian jurisdiction (Figure 3, Supplementary Figures S1–S12). Hence, those who received a dose of vaccine earlier, typically seniors and older adults, were characterized as myopic rationalists (Pearson-*r* = 0.70). This result is also consistent with studies showing a higher tendency for Canadian seniors to be socially isolated (30): When they have lower social interactions, they have less opportunity to imitate. On the other hand, the average age and employment rate were negatively highly correlated (Pearson-*r* = −0.88). So while acknowledging that correlation does not imply causation, the moderately negative correlation between the estimated proportion of rationalists and the employment rate (Pearson-*r* =−0.56) is not surprising. In addition, this correlation could also be due to lower unemployment rates in conservative provinces, such as Saskatchewan (31).

Excluding the employment rate, our results suggested no high correlation between the estimated proportion of myopic rationalists across Canada and the most important socio-economic factors such as income and the level of education as well as vaccination coverage. This is contrary to the reported results for the U.S. states (13). Although correlations do not imply causality, it could be postulated that the distribution of the decision-making strategies in the context of COVID-19 vaccination either *(i)* stems from culture rather than the most explicit socio-economic factors or *(ii)* it is driven by other factor which is impacted by the socio-economic factors and we are not aware of. The first hypothesis comes from the fact that the differences between the correlation results for Canada and the U.S. align with the arguments made by some scholars who advocate deep cultural distinctions between Canadians and Americans (32). The second hypothesis is based on the studies indicating more inclusive welfare systems in Canada compared to the U.S. and consequently a smaller income gap between Canadians compared to Americans (33).

The positive correlation between the average age and the proportion of myopic rationalists across the Canadian provinces (Pearson-*r* = 0.71) provides motivation to extend the model to an age-stratified version in future. This extension could investigate the relationship between the dominant decision-making strategy and age. For each Canadian jurisdiction, we assumed a homogeneous population sharing a same perceived excess payoff for vaccination. Yet, it has been reported that several factors, including socio-economic factors (34), existing medical conditions (35), and community characteristics (36), impact vaccine acceptance. Generalizing the model to a heterogeneous one where individuals are stratified based on these factors is left for future work.

## Data availability statement

Publicly available datasets were analyzed in this study. All data sources are cited in the article/Supplementary material. The codes are available at https://github.com/aghaeeyan/DM.

## Ethics statement

This research utilized publicly available data. No ethics approval or written informed consent was required as the study did not involve human subjects or identifiable personal information. Therefore, ethics committee approval was deemed unnecessary.

## Author contributions

AA: Conceptualization, Data curation, Formal analysis, Investigation, Methodology, Software, Validation, Visualization, Writing – original draft, Writing – review & editing. PR: Conceptualization, Funding acquisition, Investigation, Methodology, Project administration, Supervision, Writing – original draft, Writing – review & editing. ML: Conceptualization, Funding acquisition, Methodology, Project administration, Supervision, Writing – original draft, Writing – review & editing.
