# Supplementary material for: The majority of Canadians likely behaved as myopic rationalists rather than success-based learners when deciding on their first dose of COVID-19 vaccine
Source: Front Public Health. 2024 Jul 24;12:1406911. doi: 10.3389/fpubh.2024.1406911 (PMC11303299; doi:10.3389/fpubh.2024.1406911)
Supplement: Supplementary file 1 [file Data_Sheet_1.pdf]

Supplementary Information for
The majority of Canadians likely behaved as myopic rationalists rather than success-based learners when deciding on their first dose of COVID-19 vaccine

Azadeh Aghaeeyan[1*], Pouria Ramazi[1], Mark A. Lewis[2]

[1]Department of Mathematics and Statistics, Brock University, ON, Canada
[2]Department of Mathematics and Statistics and Department of Biology, University of Victoria, BC, Canada

[*]To whom correspondence should be addressed; E-mail: aaghaeeyan@brocku.ca

Estimation of the proportion of individuals concerned about the vaccine associated side effects

We used collected data during a longitudinal study conducted by Impact Canada (*1*) and estimated the proportion of individuals concerned about the vaccine associated side effects. In this longitudinal study, Canadian jurisdictions were categorized into the following six regions for the purpose of sampling:

- Manitoba, Saskatchewan, and Nunavut,
- Alberta and Northwest Territories,
- British Columbia and Yukon,
- Quebec,
- Ontario,
- Newfoundland and Labrador, New Brunswick, Nova Scotia, and Prince Edward Island.

For each region, we calculated the population proportion of individuals with concerns about vaccine associated side effects. This was done by dividing the weighted total number of responders from that region whose answers to the question “What is your top/main reason for not wanting to get a safe and/or effective COVID-19 vaccine when it becomes available and recommended” were one of the following cases: “Do not believe it will be safe”, “Will have side effects or make me sick”, “Not enough testing or research has been done”, “Do not trust the newness of the vaccine” by the weighted total number of responders from that region. This question was asked during the following five rounds of the survey:

- Wave 10–Dec 16-22, 2020,
- Wave 11–Feb 9-16, 2021,
- Wave 12–March 17-23, 2021,
- Wave 13–May 5-12, 2021,
- Wave 14–June 23-29, 2021.

We then estimated the proportion of individuals concerned about vaccine associated side-effects over time $f(t)$ using the zero order interpolation of the five calculated values for each region.

Data

We observed that as of November 21, 2021, the last date of data fitting, the numbers of vaccinated individuals in the provinces of Quebec and Nova Scotia were larger than those of June 2023, the date at which we determined the number of vaccine refusers. After investigation, it was revealed that the data managers began excluding non-residents. This adjustment was not applied to the earlier data points, though (*2*). For the provinces of Nova Scotia (and Quebec), we then multiplied all temporal data on the number of vaccinated individuals by the ratio of the number of vaccinated individuals aged 12 and above as of the first date after adjustment (April 23, 2023, and August 14, 2022, respectively) to that of the last date before adjustment (March 26, 2023, and July 17, 2022, respectively).

Supplementary Tables

In this section, we provide supplementary tables.

Table S1: The estimated parameters of the model when $c_{\bar{v}} - c_{v0}f(t)$ was replaced by a constant parameter denoted by c_v.

Jurisdiction	α_1	c_v	c_c	κ	RSS
AB	0.67	0.06	2.98E-07	0.48	1.77E+11
BC	0.69	0.07	4.02E-04	0.85	3.35E+10
MB	0.68	0.16	4.30E-03	0.58	2.47E+09
NB	0.71	0.08	2.35E-03	0.84	1.10E+09
NL	0.68	0.11	1.06E-02	0.52	5.14E+08
NS	0.80	0.11	3.8E-04	0.65	1.29E+09
NT	0.72	0.07	3.83E-04	0.27	6.98E+07
NU	0.18	0.20	5.58E-05	0.59	6.76E+06
ON	0.71	0.09	2.85E-03	0.58	3.11E+11
PE	0.78	0.10	9.84E-03	0.60	3.05E+07
QC	0.76	0.08	5.67E-04	0.65	5.15E+10
SK	0.57	0.07	2.56E-03	0.70	3.78E+09
YT	0.74	0.14	5.52E-03	0.37	2.71E+07

Table S2: The estimated parameters of the model when $c_{\bar{v}} - c_{v_0}f(t)$ was used to model the perceived benefit of vaccination in the absence of confirmed cases or deaths.

Jurisdiction	α_1	c_{v_0}	$c_{\bar{v}}$	c_c	κ	RSS
AB	0.67	0.40	0.09	3.960E-05	0.49	1.76E+11
BC	0.70	0.30	0.09	3.855E-04	0.87	3.27E+10
MB	0.70	0.01	0.10	2.208E-03	0.54	2.55E+09
NB	0.74	0.55	0.09	2.349E-03	0.75	1.06E+09
NL	0.68	0.04	0.10	3.530E-05	0.50	5.16E+08
NS	0.81	0.26	0.10	2.41E-03	0.68	1.29E+09
NT	0.72	0.21	0.10	2.969E-03	0.27	6.97E+07
NU	0.15	0.00	0.10	2.343E-01	1.05	8.21E+06
ON	0.70	0.01	0.09	2.852E-03	0.58	3.11E+11
PE	0.78	0.08	0.09	9.966E-03	0.60	3.04E+07
QC	0.79	0.89	0.10	9.21E-5	0.74	5.06E+10
SK	0.57	0.26	0.10	2.604E-03	0.66	3.76E+09
YT	0.77	0.27	0.10	9.861E-04	0.38	2.71E+07

Table S3: Estimated vaccination coverage among success-based learners.

Jurisdiction	Vaccination coverage among success-based learners
NU	0.96
MB	0.85
YT	0.79
NS	0.74
NB	0.72
BC	0.72
NL	0.72
PE	0.69
QC	0.65
ON	0.64
SK	0.60
NT	0.37
AB	0.37

Table S4: The point estimate and the lower (CI-) and upper limits (CI+) of 95% confidence interval for α.

Jurisdiction	Point estimate	CI-	CI+
AB	0.59	0.30	0.61
BC	0.64	0.61	0.66
MB	0.61	0.54	0.65
NB	0.67	0.65	0.71
NL	0.68	0.58	0.73
NS	0.70	0.63	0.72
NT	0.61	0.03	0.85
NU	0.18	0.14	0.22
ON	0.64	0.59	0.68
PE	0.74	0.69	0.78
QC	0.67	0.64	0.72
SK	0.51	0.46	0.54
YT	0.68	0.54	0.75

Table S5: The point estimate and the lower (CI-) and upper limits (CI+) of 95% confidence interval for κ.

Jurisdiction	Point estimate	CI-	CI+
AB	0.48	0.32	1.24
BC	0.85	0.75	1.37
MB	0.58	0.47	0.89
NB	0.84	0.72	1.20
NL	0.52	0.46	0.98
NS	0.65	0.50	0.9
NT	0.27	0.19	6.77
NU	0.59	0.40	0.87
ON	0.58	0.60	0.93
PE	0.60	0.56	0.91
QC	0.65	0.66	0.99
SK	0.70	0.63	3.41
YT	0.37	0.30	0.75

Table S6: The point estimate and the lower (CI-) and upper limits (CI+) of 95% confidence interval for c_v.

Jurisdiction	Point estimate	CI-	CI+
AB	0.06	0.06	0.31
BC	0.07	0.05	0.10
MB	0.16	0.12	0.28
NB	0.08	0.05	0.12
NL	0.11	0.09	0.19
NS	0.11	0.07	0.22
NT	0.07	0.07	0.98
NU	0.20	0.12	0.25
ON	0.09	0.07	0.16
PE	0.10	0.07	0.18
QC	0.08	0.06	0.14
SK	0.07	0.03	0.13
YT	0.14	0.09	0.43

Table S7: The point estimate and the lower (CI-) and upper limits (CI+) of 95% confidence interval for c_c.

Jurisdiction	Point estimate	CI-	CI+
AB	2.98E-07	3.44E-08	1.50E-03
BC	4.02E-04	5.32E-06	4.03E-04
MB	4.30E-03	7.10E-06	4.31E-03
NB	2.35E-03	6.44E-07	2.35E-03
NL	1.06E-02	5.81E-06	1.56E-02
NS	3.8E-04	1.51E-07	2.42E-03
NT	3.83E-04	2.15E-06	5.92E-03
NU	5.58E-05	0.00E+00	2.50E-01
ON	2.85E-03	7.35E-05	2.87E-03
PE	9.84E-03	2.73E-06	1.00E-02
QC	5.67E-04	6.75E-08	5.68E-04
SK	2.56E-03	2.80E-06	2.60E-03
YT	5.52E-03	1.32E-06	5.78E-03

Supplementary Figures

In this section, we present the temporal graph for each Canadian jurisdiction (Figs S1-S12). In each figure, Panel A represents the data on weekly vaccinated individuals in red, the weekly distributed vaccine doses in green, and the estimated weekly vaccinated individuals in blue. Panel B represents the estimated weekly vaccinated myopic rationalists in blue and the estimated weekly vaccinated success-based learners in red. Panel C represents the evolution of the perceived payoff gain for vaccination, $\Delta\pi(t)$, over time. Panel D represents the data on the weekly number of confirmed cases and confirmed deaths in black and magenta, respectively.

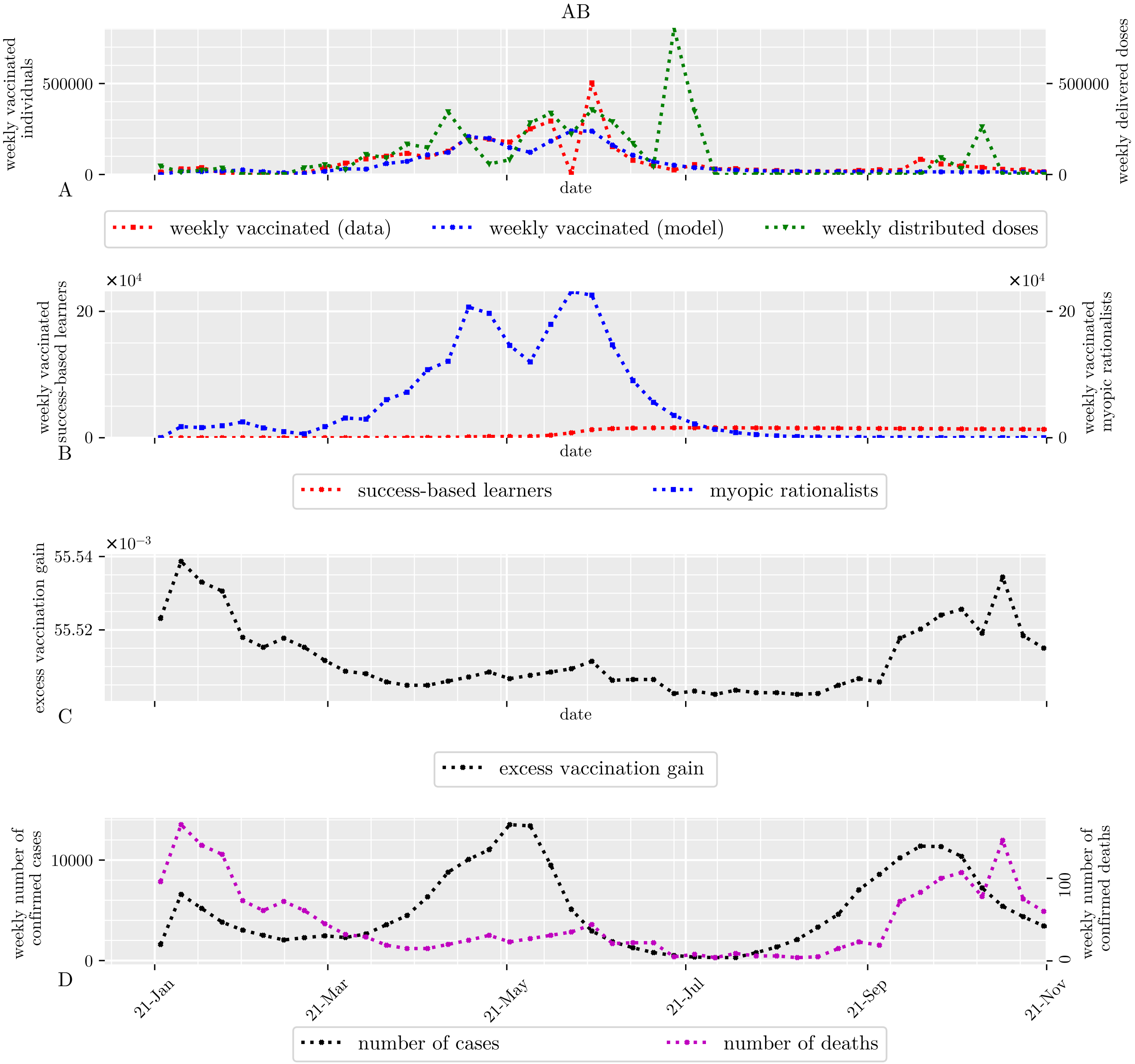

Figure S1: Alberta.

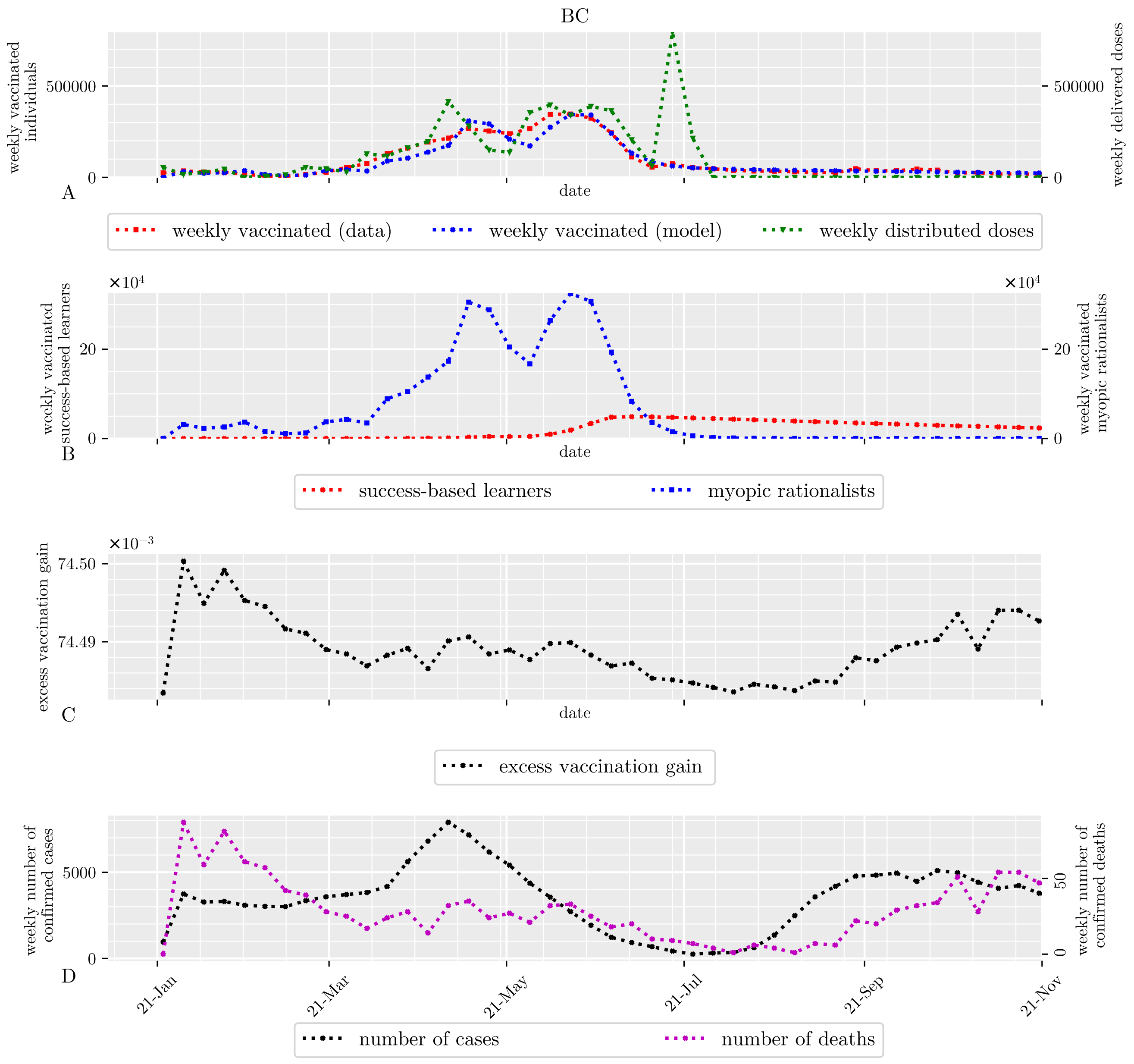

Figure S2: British Columbia

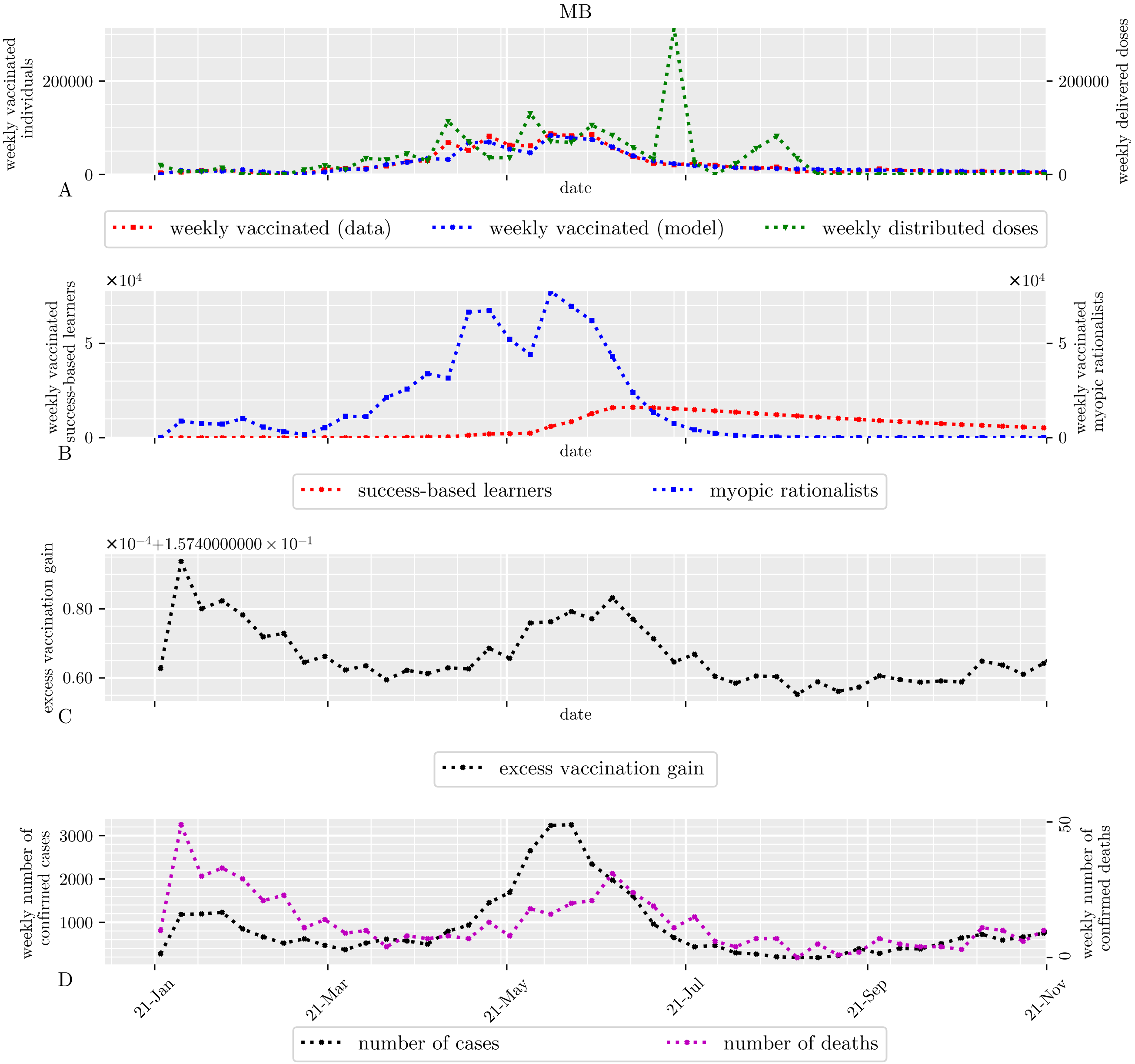

Figure S3: Manitoba.

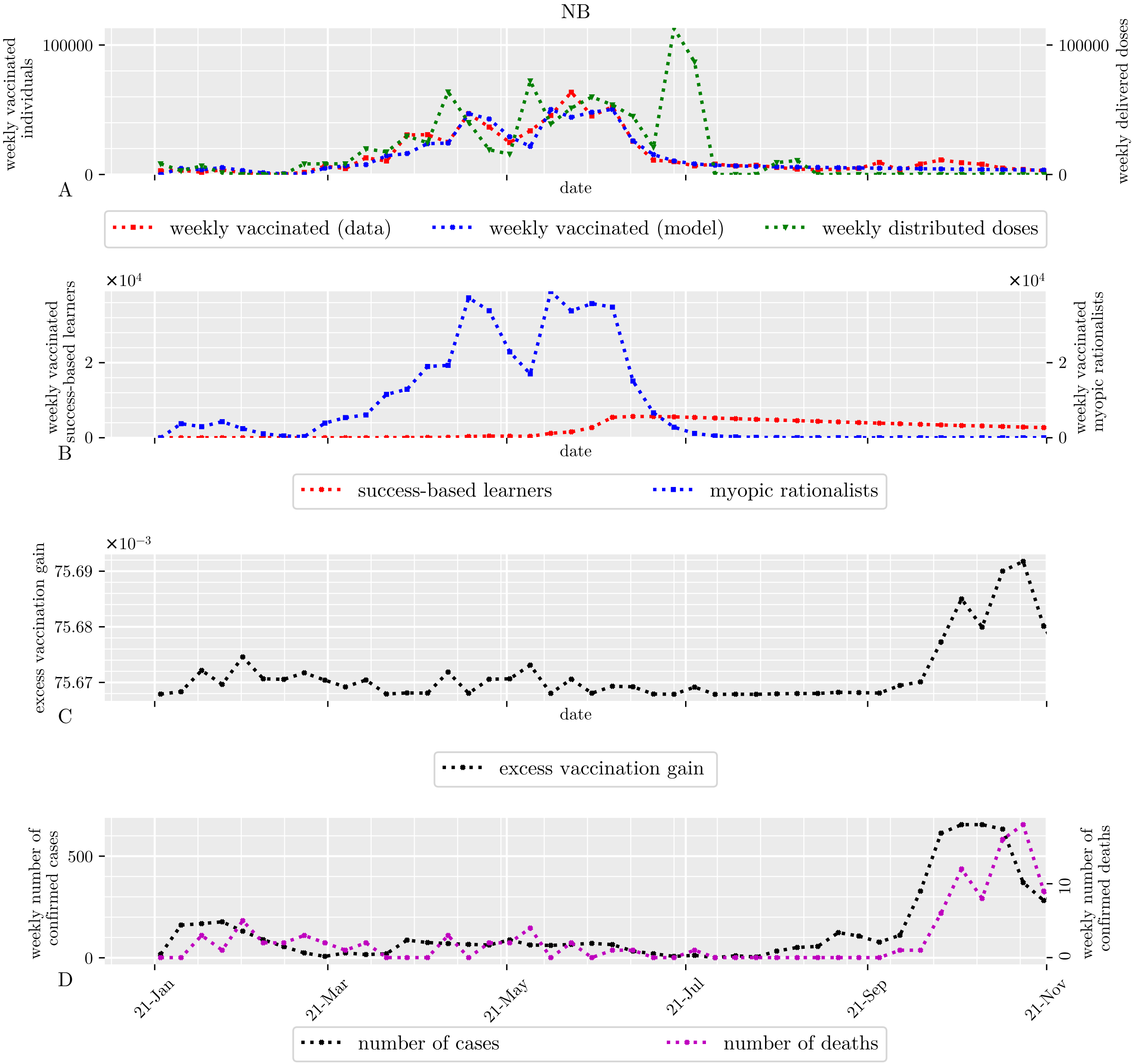

Figure S4: New Brunswick.

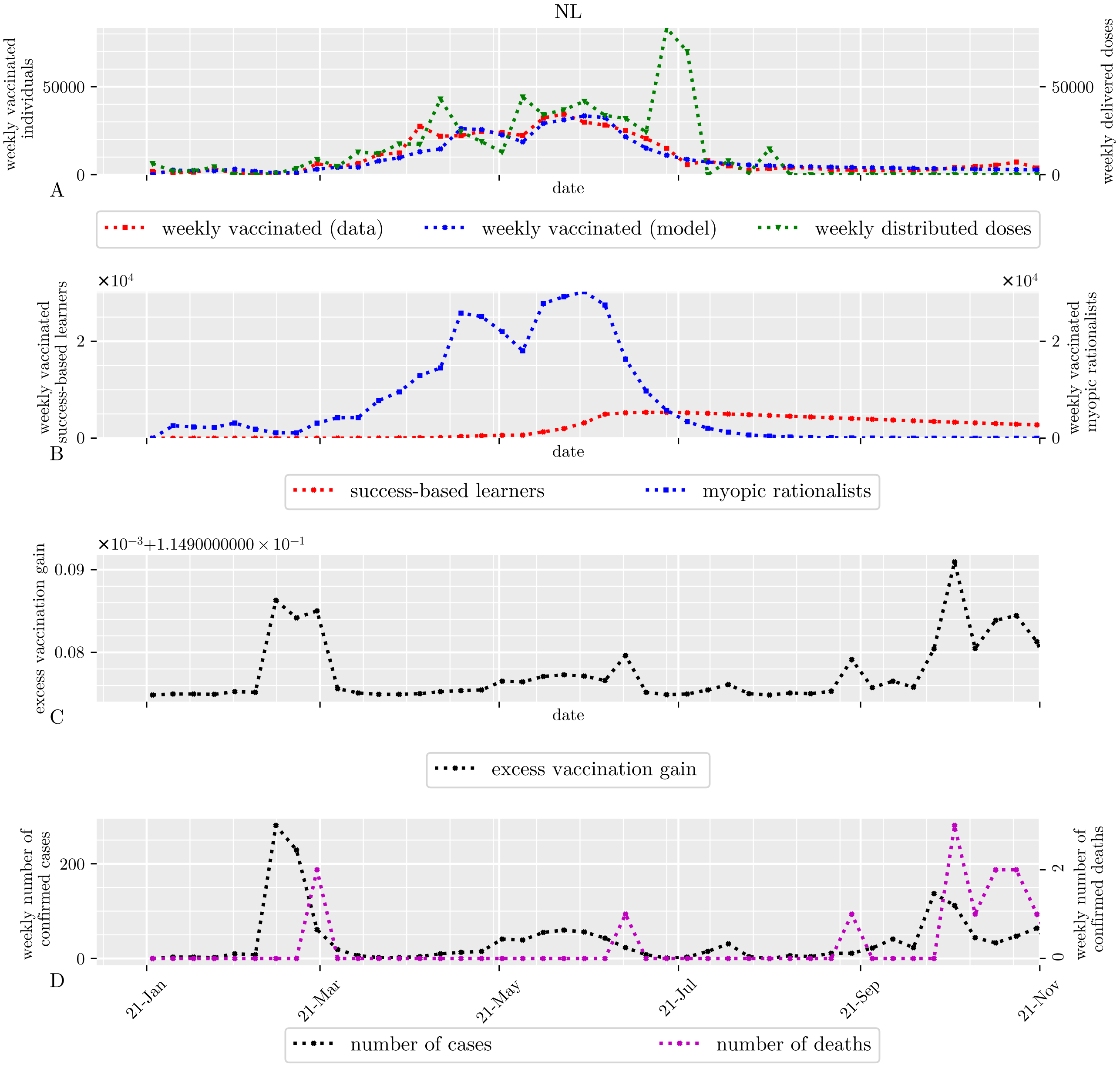

Figure S5: Newfoundland and Labrador

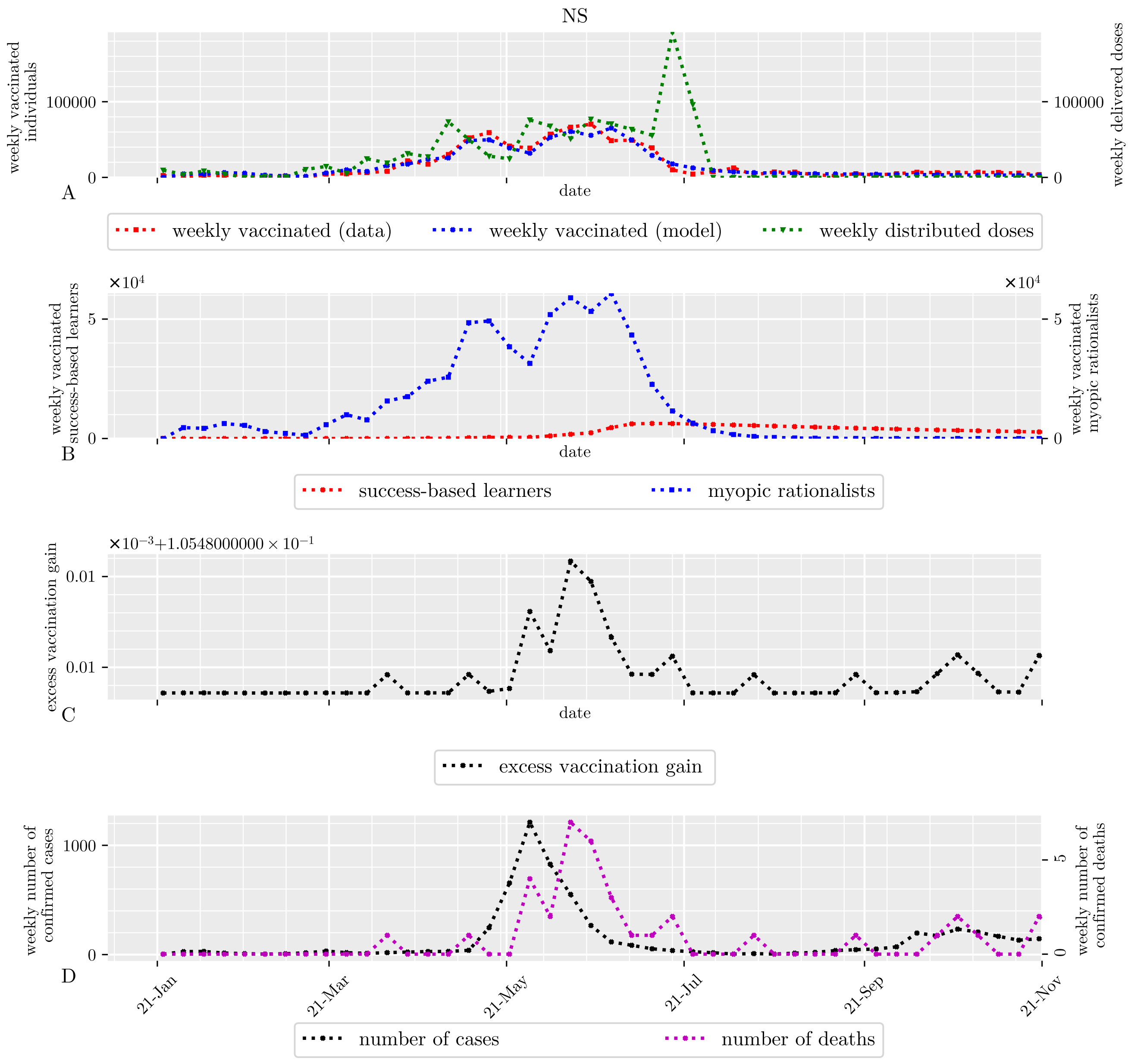

Figure S6: Nova Scotia.

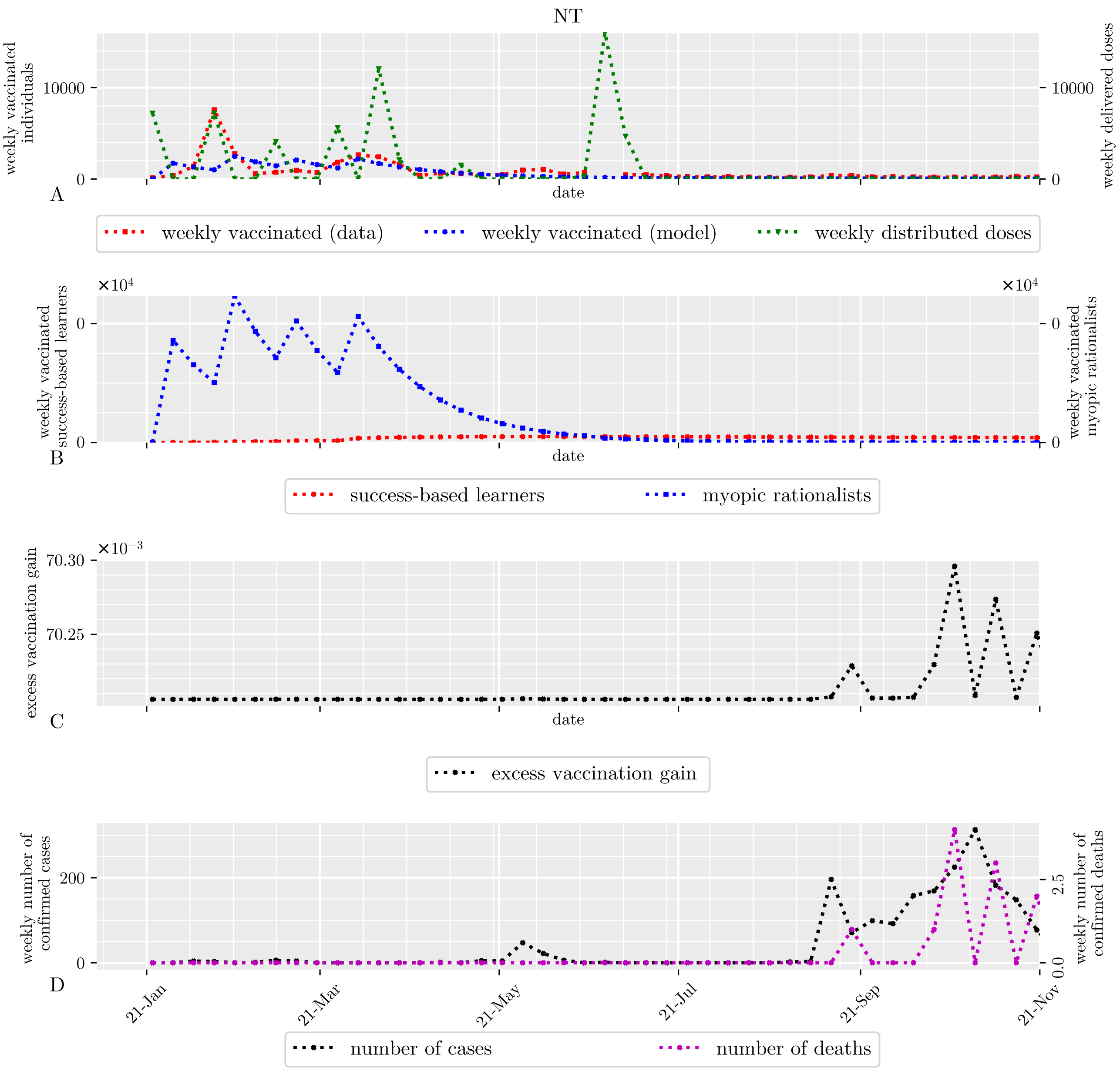

Figure S7: Northwest Territories.

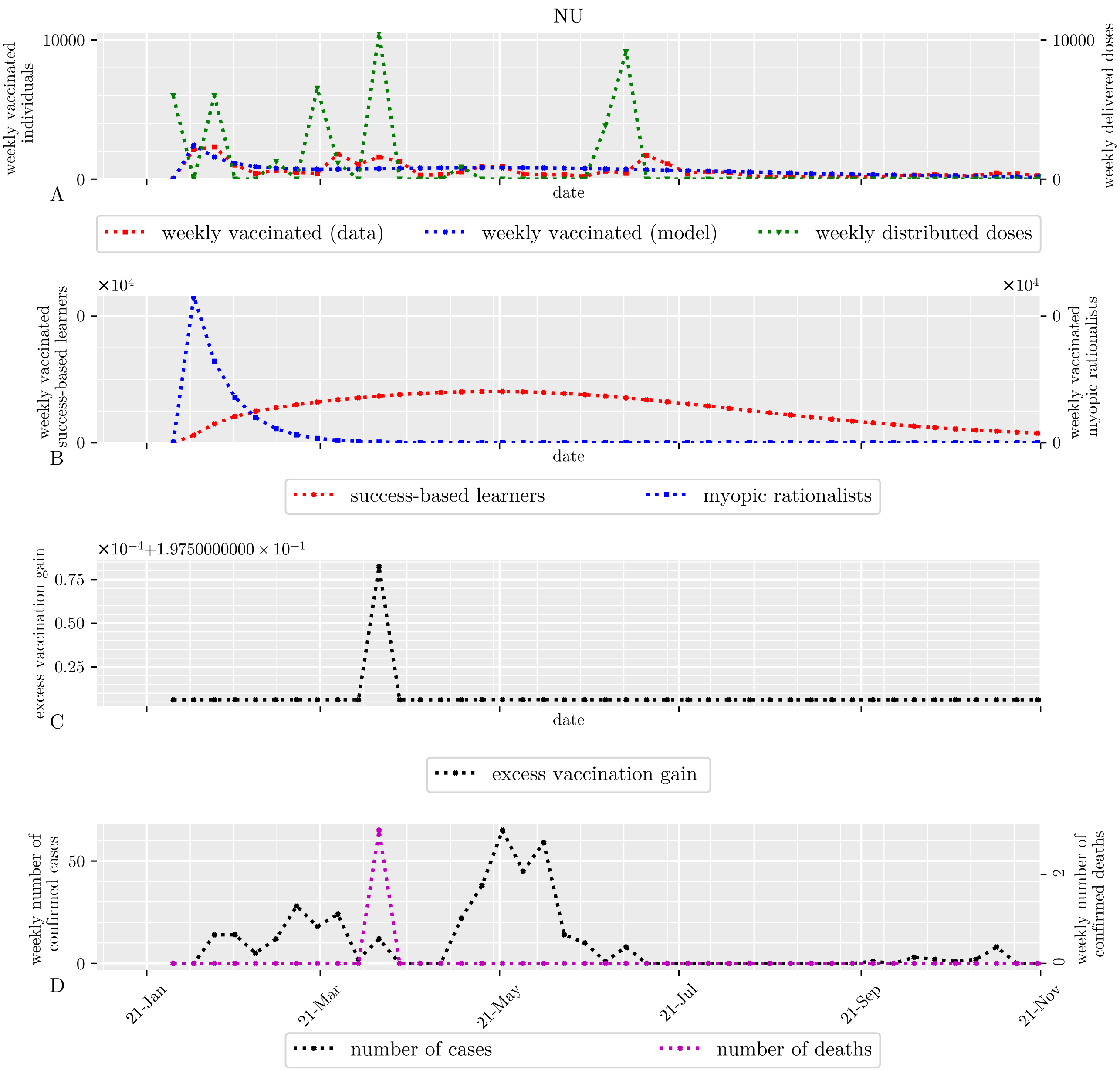

Figure S8: Nunavut.

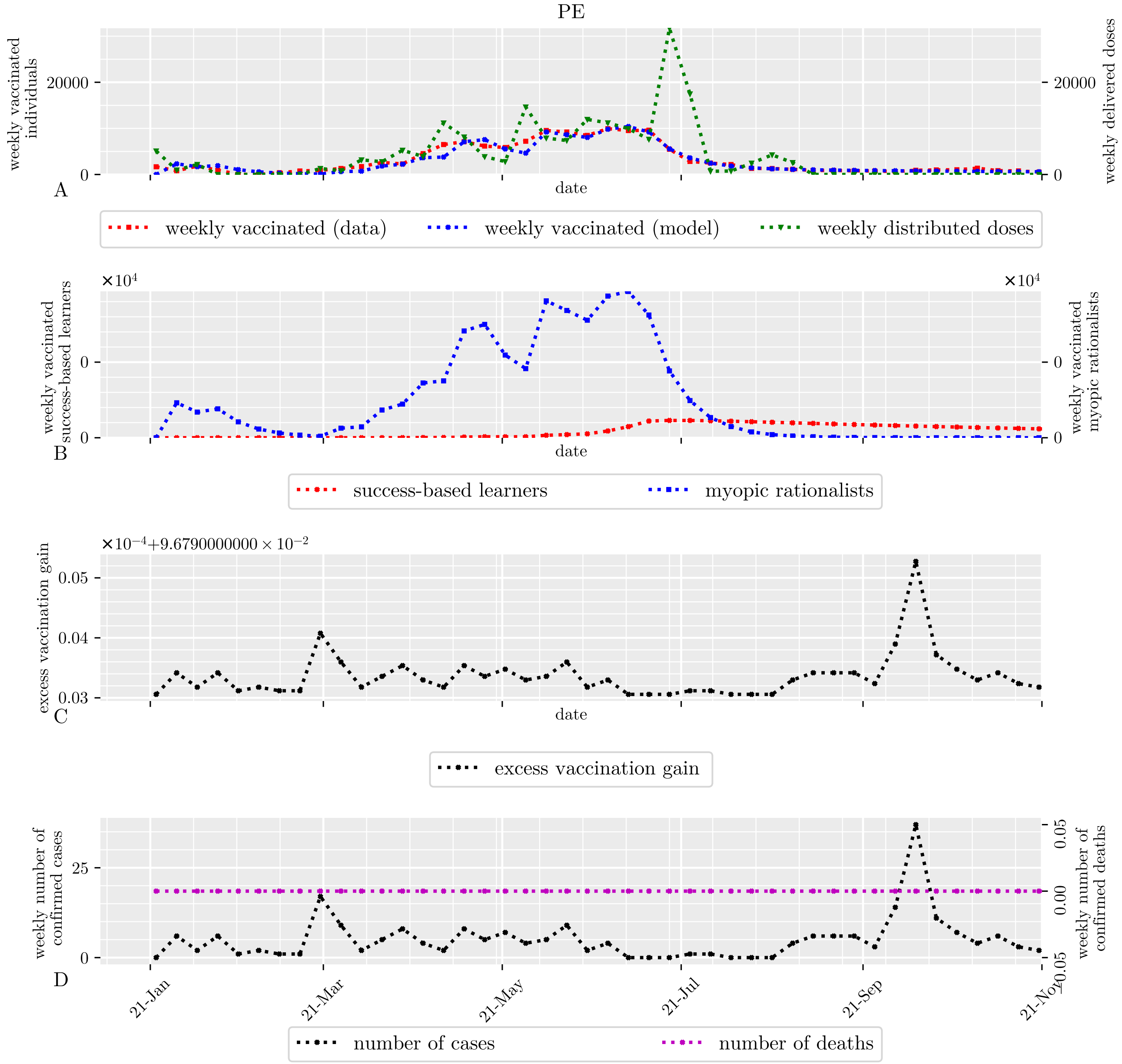

Figure S9: Prince Edward Island.

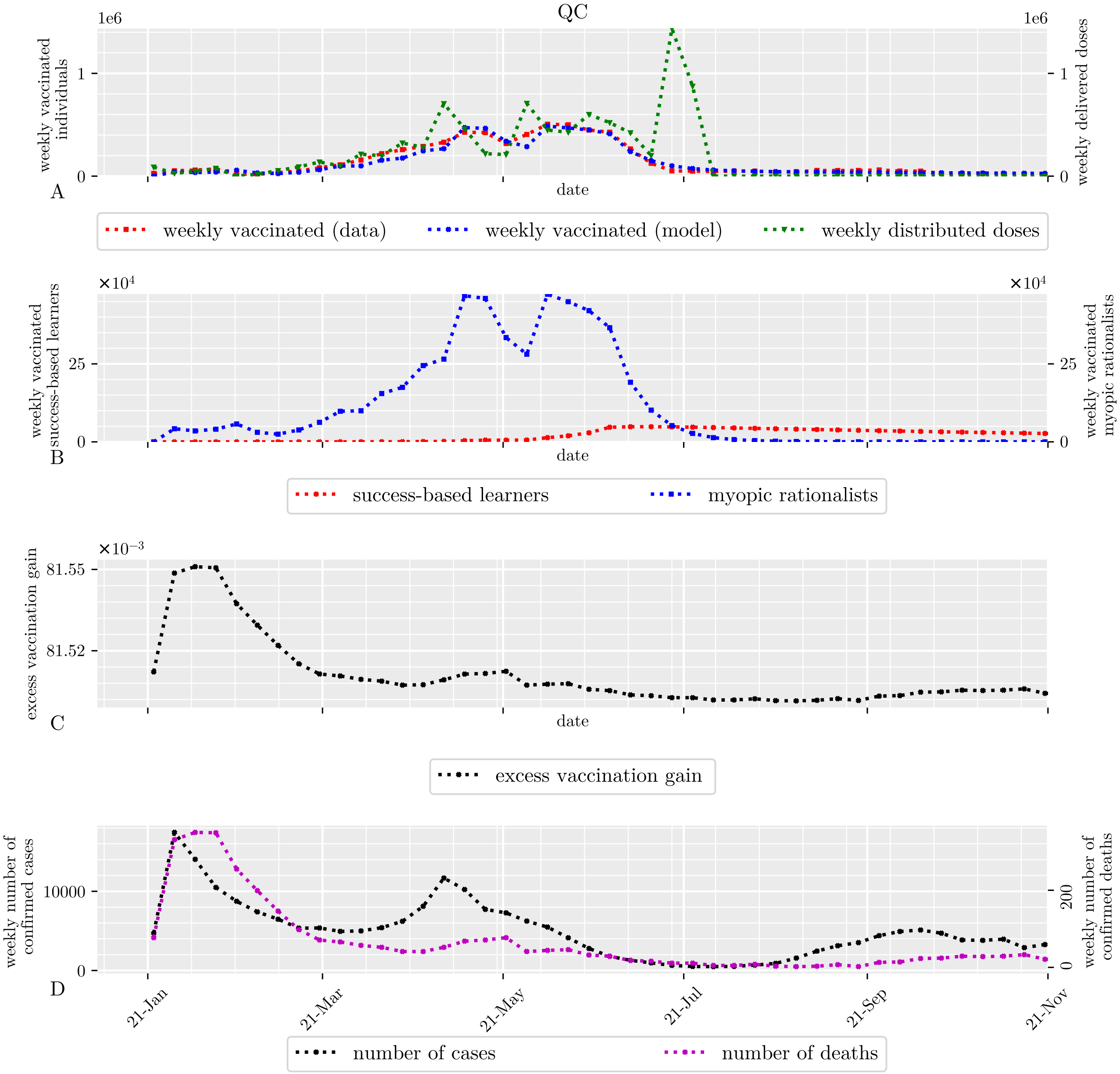

Figure S10: Quebec.

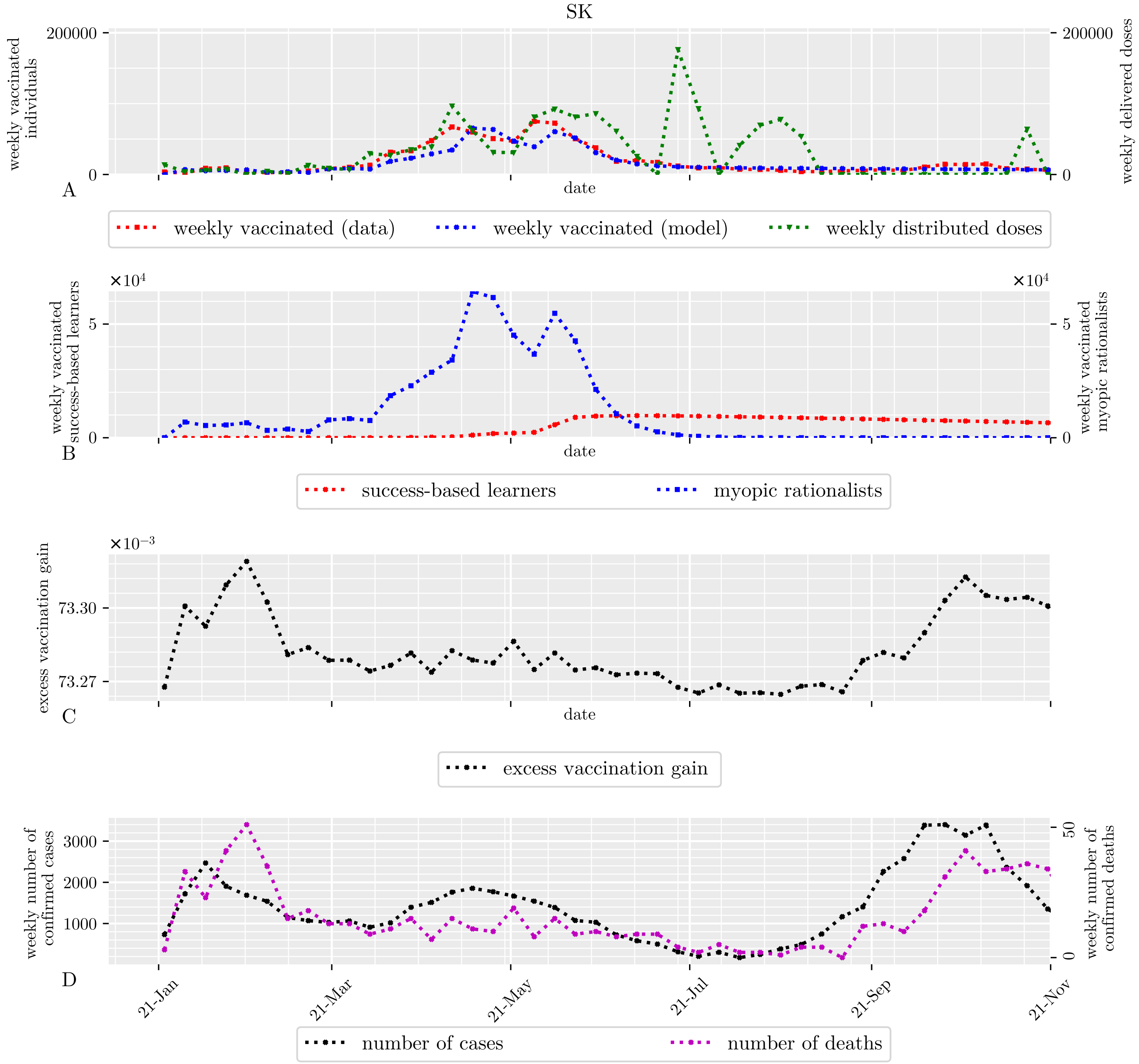

Figure S11: Saskatchewan.

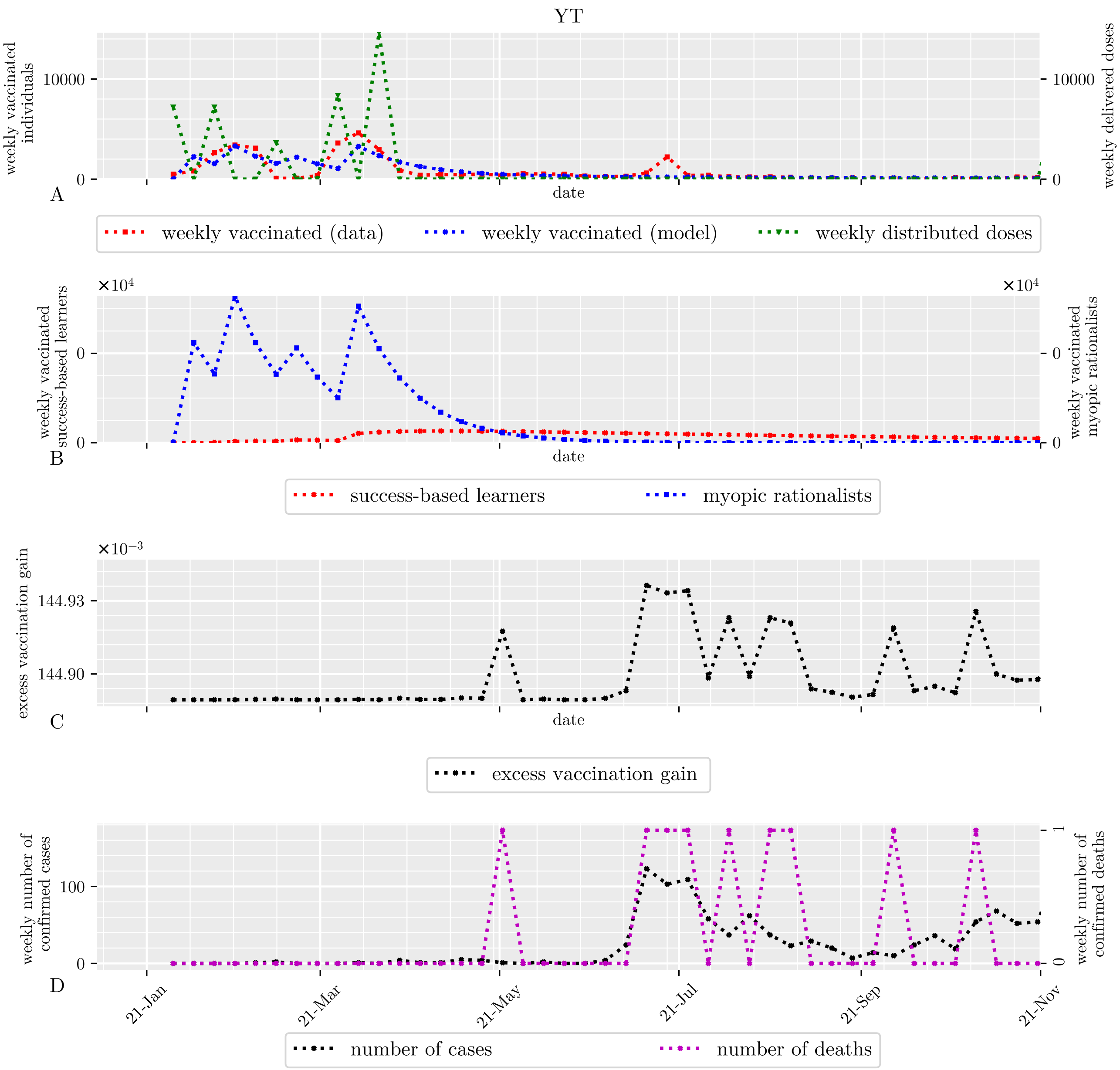

Figure S12: Yukon.

References

1. "Rapid and cost-effective monitoring of public perceptions, knowledge and behaviours - phase 2 : report. Ottawa, ON: Privy Council Office (PCO)," online, 2022, Canada. Privy Council Office, (Accessed August 2023).

2. "Canadian COVID-19 vaccination coverage report," Online, 2023, public Health Agency of Canada, (Accessed Dec 15, 2023). https://health-infobase.canada.ca/covid-19/vaccination-coverage/.
